# Uncovering a Distinct Gene Signature in Endothelial Cells Associated With Contrast Enhancement in Glioblastoma

**DOI:** 10.3389/fonc.2021.683367

**Published:** 2021-06-17

**Authors:** Fan Yang, Yuan Xie, Jiefu Tang, Boxuan Liu, Yuancheng Luo, Qiyuan He, Lingxue Zhang, Lele Xin, Jianhao Wang, Sinan Wang, Shuqiang Zhang, Qingze Cao, Liang Wang, Liqun He, Lei Zhang

**Affiliations:** ^1^ Department of Neurosurgery, Tianjin Medical University General Hospital, Tianjin Neurological Institute, Key Laboratory of Post-Neuro-injury Neuro-Repair and Regeneration in Central Nervous System, Ministry of Education and Tianjin City, Tianjin, China; ^2^ Key Laboratory of Ministry of Education for Medicinal Plant Resource and Natural Pharmaceutical Chemistry, National Engineering Laboratory for Resource Developing of Endangered Chinese Crude Drugs in Northwest of China, College of Life Sciences, Shaanxi Normal University, Xi’an, China; ^3^ Trauma Center, The First Affiliated Hospital of Hunan University of Medicine, Huaihua, China; ^4^ Precision Medicine Center, The Second People’s Hospital of Huaihua, Huaihua, China; ^5^ School of Life Science, University of Liverpool, Liverpool, United Kingdom; ^6^ Department of Neurosurgery, Tangdu Hospital of the Fourth Military Medical University (Air Force Medical University of PLA), Xi’an, China; ^7^ Department of Immunology, Genetics and Pathology, Uppsala University, Rudbeck Laboratory, Uppsala, Sweden

**Keywords:** contrast enhancement, MRI, endothelial cell, radiomics, glioblastoma

## Abstract

**Purpose:**

Glioblastoma (GBM) is the most aggressive and lethal type of brain tumors. Magnetic resonance imaging (MRI) has been commonly used for GBM diagnosis. Contrast enhancement (CE) on T1-weighted sequences are presented in nearly all GBM as a result of high vascular permeability in glioblastomas. Although several radiomics studies indicated that CE is associated with distinct molecular signatures in tumors, the effects of vascular endothelial cells, the key component of blood brain barrier (BBB) controlling vascular permeability, on CE have not been thoroughly analyzed.

**Methods:**

Endothelial cell enriched genes have been identified using transcriptome data from 128 patients by a systematic method based on correlation analysis. Distinct endothelial cell enriched genes associated with CE were identified by analyzing difference of correlation score between CE-high and CE–low GBM cases. Immunohistochemical staining was performed on in-house patient cohort to validate the selected genes associated with CE. Moreover, a survival analysis was conducted to uncover the relation between CE and patient survival.

**Results:**

We illustrated that CE is associated with distinct vascular molecular imprints characterized by up-regulation of pro-inflammatory genes and deregulation of BBB related genes. Among them, PLVAP is up-regulated, whereas TJP1 and ABCG2 are down-regulated in the vasculature of GBM with high CE. In addition, we found that the high CE is associated with poor prognosis and GBM mesenchymal subtype.

**Conclusion:**

We provide an additional insight to reveal the molecular trait for CE in MRI images with special focus on vascular endothelial cells, linking CE with BBB disruption in the molecular level. This study provides a potential new direction that may be applied for the treatment optimization based on MRI features.

## Introduction

Glioblastoma (GBM), the most aggressive and lethal type of brain tumors, is characterized by extensive vascular abnormality in both morphological and molecular levels (1). Microvascular proliferation and high vascular permeability are the hallmarks of GBM (2). Abnormal vasculature in GBM promotes tumor growth and relapses by inducing hypoxia and providing specialized niches for glioma stem-like cells (GSCs), and has been identified as a target for GBM treatment (3). MRI is a powerful non-invasive diagnostic tool for GBM and it is routinely used in clinical, providing *in vivo* portraits of tumors with multidimensional information including structure, location, composition, functional/physiological features as well as vascular parameters (4, 5). Tumor neoangiogenesis can be determined by the cerebral blood volume (CBV), which can be calculated from dynamic susceptibility contrast MRI (DSC-MRI) (6, 7). The leakiness of GBM vasculature can be detected by the conventional contrast-enhanced MRI following intravenous administration of gadolinium-based contrast agents (8). As a result of diffusion of contrast molecules out of vessels and accumulation within extracellular space in tumors, contrast-enhancing hyper-intense regions on T1-weighted (T1W) sequences are presented in nearly all GBM (9). These contrast-enhancing regions are the typical target for surgical resection (9).

Contrast enhancement (CE) is associated with distinct molecular imprints, and the plethora of radiomics studies have conclusively correlated CE with distinct molecular imprint including hypoxia signatures (10–14). However, most of these investigations were focused on tumors cells. The effect of vascular endothelial cells (ECs), the key component of blood brain barrier (BBB), on CE has not been thoroughly analyzed. In this study, we aim to understand how ECs affect CE in the tumors, and to uncover the endothelial-specific molecular imprints for CE.

## Materials and Methods

### Patients Cohorts

External cohort: 128 GBM cases (5) with publicly available transcriptome data and MRI records [The Cancer Imaging Archive (TCIA) (http://www.cancerimagingarchive.net/)] were included in this study (**Table S1**). Patients’ clinical information and processed RNA-sequencing data were obtained from the database of The Cancer Genome Atlas (TCGA) (http://cancergenome.nih.gov). The tumor segmentation information containing enhancing tumor volume (EV), central non-enhancing tumor volume (NV), complete tumor volume (CV: the sum of EV and NV) were obtained from the previous study (5).

Internal cohort: Our in-house GBM samples (14 cases) were collected at the Tangdu Hospital of the Fourth Military Medical University (Air Force Medical University of PLA) (**Table S1**). Pathological characterizations were performed according to the WHO criteria (2016). The MRI and biopsies were collected before radio- and chemotherapy, and the patients did not receive any anti-angiogenic therapy. All the patients have received corticosteroid (Dexamethasone) before surgery.

### MRI Imaging Acquisition and Preprocessing Procedures

MRI scans were performed for in-house patients with a 3.0T MRI system (MR750; GE Healthcare, Milwaukee, WI, USA) before surgery. MRI sequence included T1-weighted imaging, contrast-enhanced T1-weighted imaging, fast T2-weighted imaging, and fluid-attenuated inversion recovery imaging (FLAIR). Tumor segmentation and component volumes were analyzed according to the previous description (5). In brief, after skull-stripping with Brain Extraction Tool (BET) (15), T1-weighted images were registered to Montreal Neurological Institute (MNI) 152 standard space using the FMRIB’s Linear Image Registration Tool (FLIRT) in FMRIB software library (FSL) (16). For individual subject, all imaging modalities were co-registered to their native T1-weighted images space. MRI volumes were smoothed using Smallest Univalue Segment Assimilating Nucleus (SUSAN) to reduce intensity noise (17). Then, the automated hybrid generative-discriminative method (GLISTRboost) was used to segment the enhancing tumor volume (EV), central non-enhancing tumor volume (NV), and the complete tumor volume (CV) (18). The segmentation was confirmed by experienced neuroradiology expert and was revised if necessary.

### Immunohistochemical Analysis

Tissue sections of formalin-fixed, paraffin-embedded samples were deparaffinized and dehydrated prior to antigen retrieval followed by blocking as previously described (19). Then the sections were incubated with primary antibody towards PLVAP (HPA002279, Sigma), ABCG2 (ab24115, Abcam), and TJP1 (HPA001637, Sigma) followed by incubation with biotinylated secondary antibody and streptavidin conjugated to HRP (Vector Laboratories). The staining was developed with the DAB substrate kit (SK-4100, Vector Laboratories) according to the manufacturer’s protocol.

The images of immunohistochemical staining for VWF, CLDN5, CDH5, PECAM1, and ELTD1 in tumor were obtained from The Human Protein Atlas database (https://www.proteinatlas.org/).

### Identification of Endothelial Cell-Enriched (EC-Enriched) Genes and Gene Ontology (GO) Analysis

We used a correlation-based method (20) to identify endothelial-specific genes in the bulk transcriptome dataset from TCGA. Spearman correlation coefficients between known EC marker genes (*CDH5*, *CLDN5*, *VWF*) and all other protein coding genes were calculated. The raw P values and the False Discovery Rate (FDR) adjusted P values were also calculated. Genes with mean correlation coefficient more than 0.3 were identified as EC-enriched genes.

GO analysis for EC-enriched genes was performed using the web-based Gene Ontology tool (http://geneontology.org/), and only GO terms for biological processes were included in the analysis.

### Identification of CE-High and CE-Low Associated EC-Enriched Genes

Contrast enhancement high (CE-high) and contrast enhancement low (CE-low) tumors associated EC-enriched genes were identified by analyzing differential correlation score. In brief, according to enhancing volume/complete tumor volume ratio (EV/CV ratio), patients with top-20 and bottom-20 EV/CV ratio were selected and dichotomized into CE-high or CE-low group respectively. Correlation coefficients of EC-enriched genes to EC marker genes (*CDH5*, *CLDN5*, *VWF*) were calculated in CE-low tumor group and CE-high tumor group respectively, and then the difference between the two correlation coefficients (CE-high tumors and CE-low tumors) yields the differential correlation scores for each EC-enriched gene. Genes with differential correlation score >0.1 were classified as CE-high associated genes, whereas genes with differential correlation score < −0.4 were classified as CE-low associated genes.

Functional annotation of CE-high or CE-low associated EC genes were performed using the web-based Gene Ontology tool (http://geneontology.org/), and the GO terms with a FDR < 0.05 were considered significantly enriched.

### Survival Analysis

One hundred twenty-eight patients from TCGA database were dichotomized into CE-high or CE-low subgroups (median cutoff). Survival curves were plotted by the Kaplan-Meier method. Univariate test (log-rank) and multivariate test (Cox proportional hazards model) were used to compare survival of two subgroups.

### Statistical Analysis

Statistical analysis was performed using GraphPad Prism (GraphPad Software) and R (v 4.0.3). All the Pearson correlation coefficients analysis were performed in R with cor.test function from the stats package. The survival curves of mice and patients were analyzed by log rank test. The following p values indicate statistical significance: *p < 0.05.

## Results

### Identification of Endothelial-Enriched Genes in GBM

**Figure 1:**
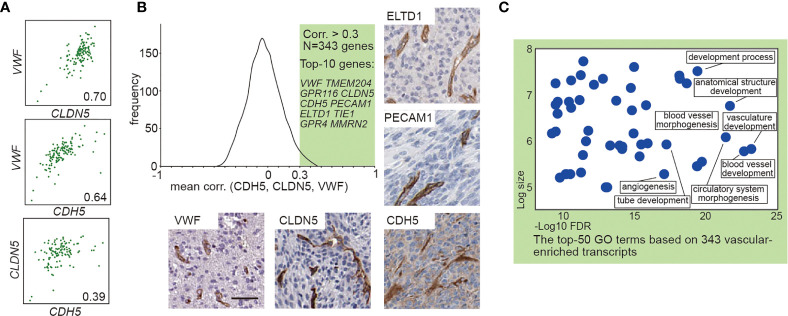
Transcript-based analysis identifies endothelial-enriched genes in GBM. **(A)** Correlation analysis between *VWF*, *CLDN5*, and *CDH5*. **(B)** Frequency distribution plot and immunohistochemical staining of 343 EC genes. The frequency distribution illustrates the distribution of the average correlation coefficients between the known EC marker (*VWF*, *CLDN5*, and *CDH5*) and the other >20,000 protein encoding genes. The immunohistochemical staining (VWF, CLDN5, CDH5, PECAM1, and ELTD1) in human GBM were obtained from Human Protein Atlas: www.hpr.se). Scale bar = 50 μm. **(C)** The enriched GO terms of the 343 EC enriched genes. The x-axis shows the enrichment statistics false discovery rate (minus log scale) and the y-axis shows the number of genes in the GO term (log scale).

To explore the effect of vascular ECs on contrast enhancement in MRI, we first identified EC-enriched genes by a systematic approach through performing the correlation analysis of the expression profiles of all gene transcripts to the known EC marker gene as previously described (20, 21). A high average correlation coefficient with those EC marker genes indicates the enrichment of the genes in EC (20, 21). We analyzed transcriptome data from 128 GBM cases with available MRI records in TCGA (**Table S1**) to produce the average correlation values between EC marker genes (*CDH5*, *CLDN5*, *VWF*) and the other >20,000 protein encoding genes, yielding 343 EC-enriched genes manifesting correlation coefficient >0.3 (**Figures 1A, B** and **Table S2**). The top 10 most highly enriched genes, including *VWF*, *TMEM204*, *GPR116*, *CLDN5*, *CDH5*, *PECAM1*, *ELTD1*, *TIE1*, *GPR4*, and *MMRN2*, are known EC enriched transcripts (21, 22). Expression of VWF, CLDN5, CDH5, PECAM1, and ELTD1 in the GBM vasculature were confirmed by immunohistochemistry (**Figure 1B**). Gene Ontology analysis of these 343 EC-enriched gene transcripts uncovered that the top significantly enriched biological process categories were all related to EC function (vasculature/blood vessel development), as well as numerous other endothelial related terms including blood vessel morphogenesis, circulatory system morphogenesis, tube development, and angiogenesis (**Figure 1C** and **Table S2**).

### Contrast Enhancement Is associated With a Distinct Molecular Signature in ECs Characterized by Upregulation of Pro-inflammatory Genes and Deregulation of BBB-Related Genes

**Figure 2:**
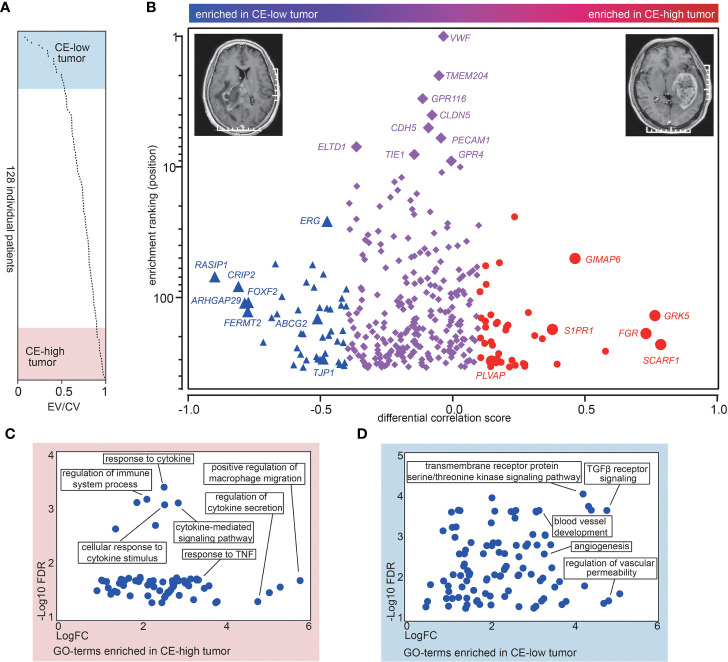
CE is associated with a distinct molecular signature. **(A)** Overview of the ratios between the enhancing volume to the complete tumor volume (EV/CV ratio) in the 128 TCGA patients. The ratios are sorted from low to high. **(B)** The differential corr. score (difference between mean corr. with CE-low and CE-high transcripts) was plotted *versus* “EC-enrichment ranking” (position of correlation coefficients categorized as 343 EC-enriched genes, highest corr. = ranking 1). The red circles represent CE-high associated genes and the blue triangles represent CE-low associated genes. **(C, D)** Gene ontology analysis of CE-high **(C)** and CE-low **(D)** associated vascular genes. The x-axis shows the enrichment fold (log scale) and the y-axis shows the false discovery rate (log scale).

To uncover the CE associated molecular signatures in vasculature, we analyzed MRI records of 128 GBM cases available in The Cancer Imaging Archive (TCIA) (https://www.cancerimagingarchive.net/) (**Table S1**). To evaluate the degree of CE for GBMs, we generated the ratio between the enhancing volume and the complete tumor volume (sum of the enhancing part and the central non-enhancing part) in the 128 TCGA GBM cases (**Figure 2A** and **Table S1**). Top- and bottom-20 patients were selected and dichotomized into contrast enhancement high (CE-high) or contrast enhancement low (CE-low) groups according to their enhancing volume/complete tumor volume ratio (EV/CV ratio) (**Figure 2A** and **Table S1**). In order to identify CE associated vascular genes, we first analyzed the correlation coefficients of the 343 EC-enriched genes to the EC markers in CE-low and CE-high groups respectively, and produced a differential correlation score (between CE-high and CE-low) for each gene. The scores indicate “degree” of EC-enrichment. High differential correlation score indicates that the gene gains EC-enrichment in CE-high tumors, which is likely due to (1) loss of expression in CE-low ECs, or (2) gain of expression in CE-high ECs. Forty-two genes with higher correlation coefficient in CE-high tumors (differential correlation score > 0.1) were classified as CE-high associated genes, including *SCARF1*, *GRK5*, *FGR*, *GIMAP6*, *S1PR1*, and *PLVAP* (**Figure 2B**, red circles; **Table S3**). On the other hand, 44 genes with higher correlation coefficient in CE-low tumors (differential correlation score < −0.4) were classified as CE-low associated genes including *RASIP1*, *CRIP1*, *ARHGAP29*, *FERMT2*, *ERG*, *FOXF2*, *ABCG2*, *TJP1*, and *COL1A2* (**Figure 2B**, blue triangles; **Table S3**). Interestingly, *TJP1* and *COL1A2* were used as markers for classical and mesenchymal subtype classification (23, 24).

Function annotation of the 42 CE-high associated vascular genes revealed significant enrichment of GO terms connected to “regulation of macrophage,” “response to cytokine/TNF,” as well as “response to cytokine secretion” (**Figure 2C**), whereas analysis of CE-low associated vascular genes uncovered GO terms including “blood vessel development,” “TGFβ receptor signaling,” and “regulation of vascular permeability” (**Figure 2D**). Taken together, these results indicate that CE are associated with alteration of genes involved in pro-inflammatory response and BBB integrity in vascular ECs.

### Increased PLVAP Expression and Decreased ABCG2 and TJP1 Expression in Vasculature in CE-High GBMs

**Figure 3:**
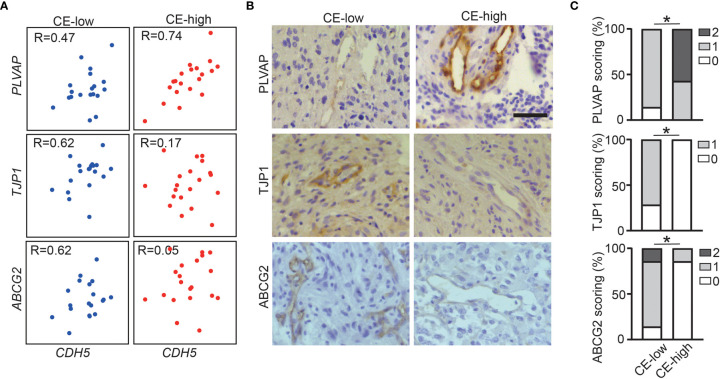
Increased PLVAP expression and decreased ABCG2 and TJP1 expression in vasculature of CE-high GBM. **(A)** Correlation plots show expression of CDH5 *versus* selected genes (*PLVAP*, *TJP1*, and *ABCG2*) in CE-low (Bottom-20 EV/CV cases in TCGA dataset) and CE-high (Top-20 EV/CV cases in TCGA dataset). **(B)** Immunohistochemistry staining of PLVAP, TJP1, ABCG2 in CE-low and CE-high groups. **(C)** Quantification of immunohistochemistry staining of PLVAP, TJP1, ABCG2 in CE-low and CE-high groups. Staining was scored semi-quantitatively on scale from 0 to 2 (0, no vessels stained; 1, minority of vessels stained; and 2, majority of vessels stained) (Mann-Whitney test, *p < 0.05). Scale bar = 50 μm.

To validate our findings showing the association of CE with deregulation of BBB related genes in the GBM vasculature, we performed immunohistochemical staining for PLVAP, ABCG2, and TJP1 on in-house CE-high or CE-low GBM cases. All three proteins had vascular staining patterns (**Figures 3A–C**). As expected, PLVAP was up-regulated in the vasculature in CE-high GBMs, while ABCG2 and TJP1 were upregulated in the vasculature of CE-low GBMs (**Figures 3A–C**).

### Contrast Enhancement Is Associated With Poor Prognosis and Mesenchymal Subtype

**Figure 4:**
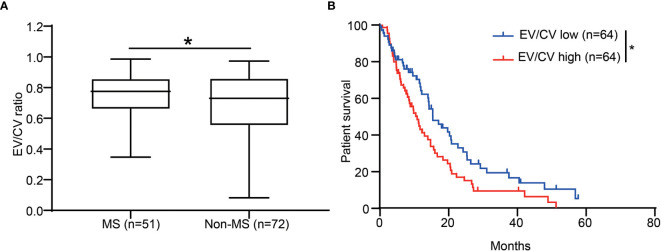
CE was associated with patient survival and enriched in the GBM mesenchymal subtype. **(A)** The ratio of EV/CV was enriched in mesenchymal subtype. Student’s t-test, *p < 0.05 **(B)** Kaplan-Meier graph showing patient survival in EV/CV low and EV/CV high groups. Log-rank test, *P < 0.05.

The identification of the association of CE with pro-inflammation led us to further investigate CE in different molecular subtypes of GBM. It has been shown that mesenchymal subtype was the most pro-inflammatory subtype of GBM, which associated with higher immune-associated signaling pathways and immune cells infiltration compared to other non-mesenchymal subtypes including pro-neural and classical subtypes (25, 26). As expected, EV/CV ratio was significantly higher in mesenchymal subtypes (**Figure 4A**). The association of CE-high phenotype with mesenchymal subtype was supported by Kourosh’s study with 43 patients (27).

We next set out to assess whether CE correlates with patient survival. By dichotomizing patients into two groups of equal size according to EV/CV ratio, we found that the CE-high group was associated with shorter survival (**Figure 4B**, P = 0.0211, log-rank test). In addition, association of high contrast enhancement with poor prognosis was observed in younger patients (<60 years old) but not in older patients (≥ 60 years old) (**Figures S1A, B**). CE did not significantly correlate to the survival when correcting for age in a multivariate analysis (**Table S4**), indicating CE is not an independent prognostic marker.

## Discussion

It has been shown in several studies that CE is associated with inter- and intro-tumoral molecular signature in GBM (10–14). By comparing gene expression in specimen from 22 incompletely contrast-enhancing and 30 completely contrast-enhancing untreated glioblastoma, it revealed that CE was associated with distinct transcriptome signature characterized by increased VEGFA expression (10). These results were supported by Diehn et al. study with 22 GBM patients revealing a strong association between CE and hypoxia signature including VEGFA (11). In addition, with biopsies derived from distinct tumor regions by MRI-guided stereotactic sampling techniques, intro-tumoral heterogeneity of CE was investigated (12–14). Transcriptome analysis of biopsy from paired enhancing and peri-tumoral non-enhancing region from 13 treatment-naïve GBM patients indicated that enhanced regions were characterized by increased level of hypoxia, cellular density, and vascular hyperplasic together with elevated relative cerebral blood volume (CBV) and reduced apparent diffusion coefficient (ADC) (12, 13). Similarly, Van Meter et al. studied the molecular profiles of contrast enhancing region and central non-enhancing necrotic region and showed an enrichment of angiogenesis and hypoxia signature in central non-enhancing necrotic region (14).

In present study, we have integrated public dataset with our in-house patient cohort to provide additional molecular trait for CE with special focus on vascular ECs. ECs are key component of BBB, controlling the vascular permeability and leakiness (28). However, the direct molecular imprint of CE in ECs has not been studied before. Here, we used an unbiased approach to identify CE-low and CE-high associated EC-enriched genes. In contrast to direct comparison, this correlation-based method allows identification cell-type-enriched transcriptome using bulk RNA-seq data. We demonstrated that CE-high in GBM are associated with upregulation of pro-inflammatory genes and deregulation of BBB related genes in EC. The results were supported by the previous study with 148 GBM cases revealing a strong association of CE with an elevated inflammatory response (29).

Alteration of BBB-related genes in CE-high GBM vasculature is noteworthy. In the present study, we provide evidence in the molecular level linking CE with BBB alteration in EC characterized by up-regulation of PLVAP and down-regulation of TJP1 and ABCG2 in vasculature of CE-high GBMs. Plasma lemma vesicle-associated protein (*PLVAP*) is a vascular marker of BBB disruption, and can be induced in vasculature and associated with vascular leakage (30). In normal physiological condition, PLVAP expression is only restricted to vasculature in choroid plexus and circumventricular organs where the ECs are fenestrated to allow exchange between blood and cerebrospinal fluid (CSF) (31). PLVAP could increase vascular permeability by promoting transcytosis in ECs through forming the diaphragms of caveolae, fenestrae, and trans-endothelial channels (31). TJP1, also known as ZO-1, is essential for tight junction formation (32, 33). At BBB, TJP1 link the claudins and occludins to the actin cytoskeleton, sealing ECs (32, 33). *ABCG2* encode breast cancer resistance protein (BCRP), which is the ATP-binding cassette transporter mediating efflux of xenobiotics including temozolomide and other low molecular weight anti-cancer drugs from the endothelium away from the neuro parenchymal space (34). The deregulation of these important proteins indicates that CE may serve as an imaging biomarker for BBB disruption. Interestingly, steroids and anti-angiogenic therapy play an essential role on vascular permeability, and how the steroid and anti-angiogenic treatments affect ECs leading to CE alteration deserves further investigation.

In conclusion, we have shown that CE in GBM was associated with BBB alterations in vascular ECs. Considering the established key role of BBB on systemically delivery of pharmacological agents into the brain, whether CE is associated with drug delivery and could be a non-invasive image biomarker for monitoring the drug delivery deserves further investigation. Considering the established key role of BBB on systemical delivery of pharmacological agents into the brain, our results support further research to develop CE as a potential non-invasive image biomarker for predicting drug delivery in the future.

## Conclusion

Our study provided additional insights to reveal molecular trait for CE in MRI images with special focus on vascular ECs in glioblastoma. We demonstrated that high CE was associated with distinct gene signatures characterized by deregulation of BBB-related genes and up-regulation of pro-inflammatory genes.

## Data Availability Statement

The datasets presented in this study can be found in online repositories. The names of the repository/repositories and accession number(s) can be found in the article.

## Ethics Statement

The studies involving human participants were reviewed and approved by the Ethics Committee of Shaanxi Normal University. The patients/participants provided their written informed consent to participate in this study.

## Author Contributions

LZ, LH, and LW conceived the project. FY, YX, JT, and BL performed the experiments, YL, QH, LXZ, LX, JW, SW, SZ, and QC analyzed data. LZ, LH, and LW wrote the manuscript with significant input from FY and YX. All authors contributed to the article and approved the submitted version.

## Funding

This work was supported by the National Natural Science Foundation of China (NSFC)/the Swedish Foundation for International Cooperation in Research and Higher Education (STINT) Mobility Program (No. 81911530166), the NSFC (No. 81702489, 82002659, 81870978, 81772661), the National Key R&D Program of China (No. 2018YFC1313003), the Natural Science Foundation of Shaanxi Province (No. 2021KW-46, 2020JQ-429, 2020JZ-30), Tianjin Natural Science Foundation (No. 18JCYBJC94000), the Natural Science Foundation of Hunan Province (No. 2020JJ4071), Fundamental Research Funds for the Central University (No. GK202003050, GK202003048), the Natural Science Foundation of Huaihua City (2020R3118, 2020R3116).

## Conflict of Interest

The authors declare that the research was conducted in the absence of any commercial or financial relationships that could be construed as a potential conflict of interest.

## Supplementary Material

Supplementary Table 1 Patients’ information

Supplementary Table 2 Endothelial enriched genes identified by correlation analysis

Supplementary Table 3 Contrast enhancement associated endothelial enriched genes

Supplementary Table 4 Multivariate survival analysis with contrast enhancement
