# Supplementary material for: Uncovering a Distinct Gene Signature in Endothelial Cells Associated With Contrast Enhancement in Glioblastoma
Source: Front Oncol. 2021 Jun 17;11:683367. doi: 10.3389/fonc.2021.683367 (PMC8245778; doi:10.3389/fonc.2021.683367)
Supplement: Supplementary Table 4 — Multivariate survival analysis with contrast enhancement [file Table_4.docx]

**Table S4 Multivariate survival analysis with contrast enhancement (EV/CV ratio)**

| **Covariate** | **b** | **SE** | **Wald** | **P** | **Exp (b)** | **95% CI of Exp (b)** |
| --- | --- | --- | --- | --- | --- | --- |
| **Age** | 0.03554 | 0.009726 | 13.3514 | 0.0003 | 1.0362 | 1.0166 to 1.0561 |
| **EV/CV ratio** | 0.5438 | 0.6105 | 0.7934 | 0.3731 | 1.7225 | 0.5206 to 5.6995 |

b, regression coefficient; SE, standard error
