# Supplementary figures and images for: Uncovering a Distinct Gene Signature in Endothelial Cells Associated With Contrast Enhancement in Glioblastoma
Source: Front Oncol. 2021 Jun 17;11:683367. doi: 10.3389/fonc.2021.683367 (PMC8245778; doi:10.3389/fonc.2021.683367)

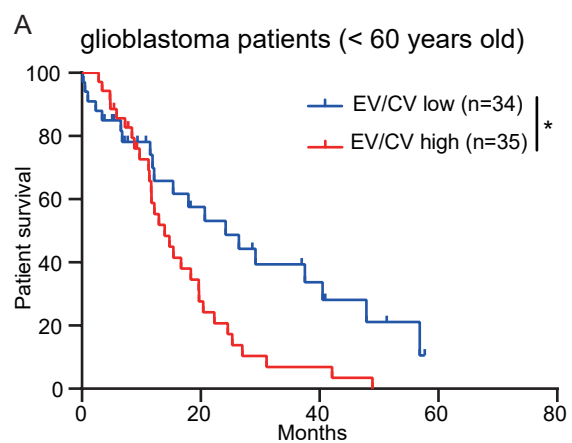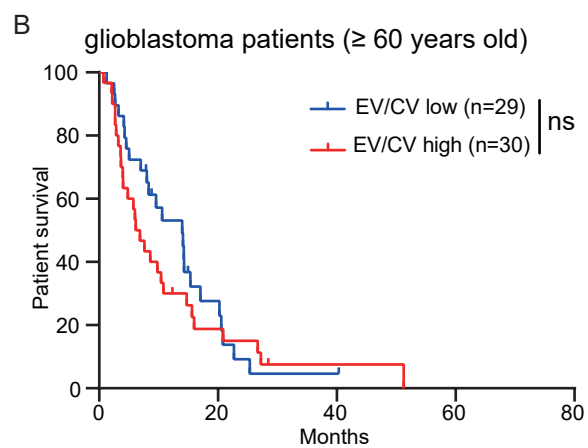

Supplement: Supplementary file 5 [file Image_1.pdf]
